# Unlocking rivers' hidden diversity and ecological status using DNA metabarcoding in Northwest Spain

**DOI:** 10.1002/ece3.70110

**Published:** 2024-08-01

**Authors:** Álvaro Fueyo, Omar Sánchez, Carlos Carleos, Amando Escudero, Javier Cordón, Javier Granero‐Castro, Yaisel Juan Borrell

**Affiliations:** ^1^ Environment and Sustainability Area, Taxus Medio Ambiente Oviedo Spain; ^2^ Department of Functional Biology, Genetics University of Oviedo Oviedo Spain; ^3^ Department of Organisms and Systems Biology, Zoology University of Oviedo Oviedo Spain; ^4^ Department of Statistics and Operations Research and Mathematics Didactics University of Oviedo Oviedo Spain

**Keywords:** bulk samples, ecological status, eDNA, exotic species, IBMWP, macroinvertebrates

## Abstract

Rivers are crucial ecosystems supporting biodiversity and human well‐being, yet they face increasing degradation globally. Traditional river biomonitoring methods based on morphological identification of macroinvertebrates present challenges in terms of taxonomic resolution and scalability. This study explores the application of DNA metabarcoding analysis in both bulk and environmental DNA (eDNA) samples for comprehensive assessment of macrozoobenthic biodiversity, detection of invasive and endangered species, and evaluation of river ecological status in northwestern Spain. DNA metabarcoding of homogenized bulk samples and water eDNA revealed a mean of 100 and 87 macrozoobenthos species per sample respectively. However, the specific composition was significantly different with only 27.3% of the total species being shared. It was not possible to identify all the OTUs to species level; only 17.43% and 49.4% of the OTUs generated could be identified to species level in the bulk and eDNA samples, respectively. Additionally, a total of 11 exotic species (two first records for the Iberian Peninsula and another three first records for Asturias region) and one endangered species were detected by molecular tools. Molecular methods showed significant correlations with morphological identification for EQR values (Ecological Quality Ratio) of IBMWP index, yet differences in inferred river ecological status were noted, with bulk samples tending to indicate higher status. Overall, DNA metabarcoding offers a promising approach for river biomonitoring, providing insights into biodiversity, invasive species, and ecological status within a single analysis. Further optimization and intercalibration are required for its implementation in routine biomonitoring programmes, but its scalability and multi‐tasking capabilities position it as a valuable tool for integrated monitoring of river ecosystems.

## Graphical abstract

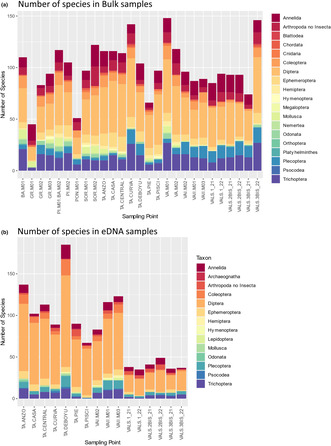

## 1 INTRODUCTION

Fluvial ecosystems play a vital role in supporting biodiversity, providing freshwater, and sustaining human societies (Bunn & Arthington, 2002; Dudgeon et al., 2006). Over 2 billion people directly rely on rivers for their water needs; at the same time, these environments host an abundance of life, including more than half of all fish and at least 10% of all known species, highlighting their critical importance (WWF, 2024). Despite their significance, global rivers face increasing degradation, posing challenges to both ecological integrity and human well‐being (Millennium Ecosystem Assessment, 2005). In this context, many European water bodies face significant challenges in achieving and maintaining good ecological status. According to the European Environment Agency (EEA), only 40% of European rivers are currently in good ecological condition, highlighting the urgent need for comprehensive monitoring and conservation efforts (European Environmental Agency, 2018). The Water Framework Directive (WFD‐2000/60/EC) of the European Union, for instance, underscores the importance of achieving and maintaining good ecological status in surface waters, including rivers, by assessing the abundance and diversity of aquatic organisms. Consequently, understanding and monitoring the biodiversity within river ecosystems have become fundamental measure of their ecological health and compliance with regulatory standards. Macrozoobenthos, which include a variety of benthic invertebrates such as arthropods, annelids, platyhelminthes, and mollusks, are an important part of river ecosystems. These organisms contribute significantly to nutrient cycling, sediment dynamics, and serve as key indicators of environmental quality (Allan et al., 2021). The study of macrozoobenthic biodiversity provides insights into the overall health of river ecosystems, making it an essential aspect of ecological research and conservation efforts. Biomonitoring serves as a powerful tool for assessing the ecological condition of rivers, providing valuable insights into the impacts of human activities on aquatic ecosystems. Traditionally, the assessment of river health has heavily relied on biomonitoring approaches with macroinvertebrates serving as bioindicators due to their sensitivity to environmental changes (Furse, Hering, Moog, et al., 2006). Morphological identification of macroinvertebrates has been the conventional method employed in biomonitoring programs, where taxonomic expertise is crucial for accurate species determination. However, this approach has inherent limitations, including time‐consuming processes and the requirement for specialized taxonomic knowledge, hindering large‐scale and efficient monitoring (Bush et al., 2019). In recent years, advancements in molecular biology have introduced innovative tools such as DNA metabarcoding and environmental DNA (eDNA), offering promising alternatives to traditional morphological identification (Deiner et al., 2017; Liu et al., 2019). In the context of river ecosystems, the application of DNA metabarcoding and eDNA methods provide a scalable and highly sensitive approach for assessing, not only macrozoobenthic but also global freshwater river diversity. One of the major advantages is the scalability of DNA metabarcoding, which allows for the simultaneous analysis of multiple samples, which is particularly useful for large‐scale monitoring programs, providing a comprehensive insight into the biodiversity of river ecosystems (Ficetola & Taberlet, 2023). Moreover, molecular techniques facilitate the identification of taxa to species level, overcoming challenges associated with the time–cost limitation of the morphological identification. This species‐level identification potentially allows the data obtained to also be used for detecting species of interest, whether they are exotic and invasive species or endangered and protected species, without further effort or cost. Therefore, this multitask approach would increase the suitability of DNA metabarcoding for routine river biomonitoring.

This study aims first to apply DNA‐based molecular techniques, specifically DNA metabarcoding and environmental DNA (eDNA) analysis, for a comprehensive assessment of macrozoobenthic‐specific biodiversity and river ecological status in northwestern Spain. Moreover, since the metabarcoding procedures will be done using the generalist COI gene and much more than only macrozoobenthic species can be detected, this multitask approach would help also provide valuable insights about the presence of other species relevant to river management (i.e., invasive and/or protected species) widening the spectrum on DNA metabarcoding potentialities for future biomonitoring strategies in Spain in a more holistic and effective framework.

## 2 MATERIALS AND METHODS

### 2.1 Samplings

**FIGURE 1:**
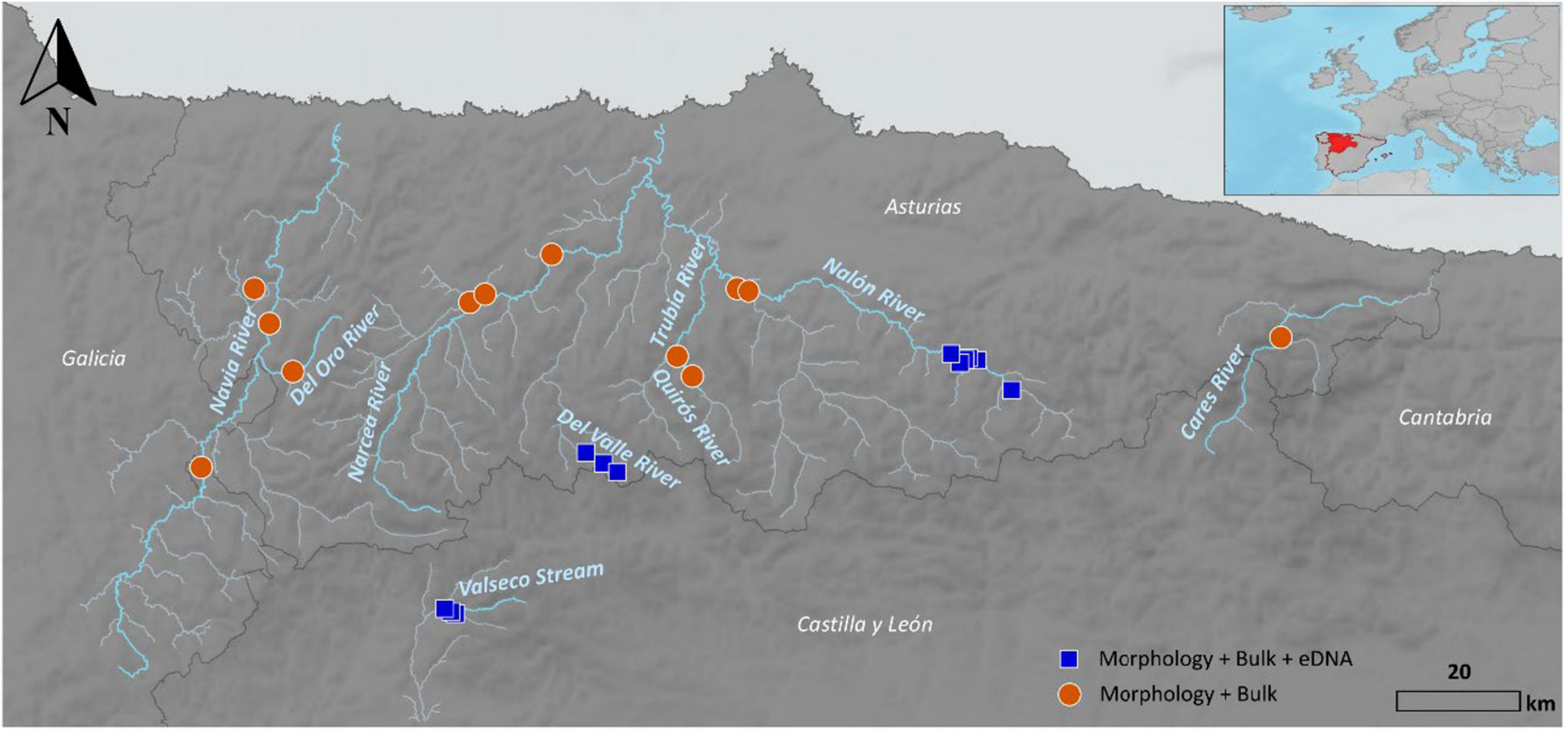
Map showing sampling locations in rivers from Northwest Spain. The colors and shapes vary depending on the sampling technique applied at each locality: Orange Circles: Morphological identification and bulk metabarcoding; Blue Squares: Morphological identification, bulk metabarcoding, and eDNA metabarcoding.

A total of 27 sites of the NW of the Iberian Peninsula were sampled for this study, taking in parallel kick samples for morphological analysis and bulk samples for metabarcoding. At 16 of these sites, water samples were also collected for eDNA metabarcoding analysis (Figure 1). Kick samples and bulk samples were collected following the sampling protocol of the Spanish Ministry of the Environment (MAGRAMA, 2013a) which consists of a semiquantitative, stratified, and multihabitat sampling scheme with a 500 μm pore Surber net and then preserved in 96% ethanol with 0.01% BAC (benzalkonium chloride). All materials contacting bulk samples were sterilized before each sampling with 10 times diluted commercial bleach (final concentration of 0.4% Cl) and washed twice with distilled water. For eDNA sampling, a transect was set up perpendicular to the river, with 5 one‐liter water samples collected across the width of the river and taken close to the river bottom. Samples from each point were pooled in a 5‐liter bottle to which 1 mL of 50% BAC was added and stored, no more than 24 h, at 4 degrees until extraction (Jo et al., 2021; Takahara et al., 2020; Yamanaka et al., 2017).

### 2.2 DNA extractions

Kick samples for morphological identification were processed according to the Spanish official protocol (MAGRAMA, 2013a). Briefly, it consists of a sieving process that separates the sample into three sieves (5, 1, and 0.5 mm pore size), and then a portion of the individuals present in each sieve is randomly sub‐sampled and a minimum of 100 individuals from each sieve are identified under the magnifying glass. Only the taxonomic groups listed in the protocol are considered for identification. In terms of resolution, the identification of macroinvertebrate taxa is multilevel because it includes identifications at different taxonomic levels, with the family level being the most common (1 genus, 121 families, 1 superorder, 1 subclass, 1 class). Bulk samples were elutriated with distilled water to remove stones and sand and then homogenized according to the protocol of Buchner et al. (2021) consisting of grinding the elutriated sample in a blender, ensuring an effective decontamination between the different samples. Homogenized bulk samples were extracted twice (10 g per replicate), in addition to two negative controls, using the MaxPowerSoil Kit (Qiagen) following the manufacturer's instructions.

Water eDNA samples were filtered through a 0.45 μm CN filter in triplicate (1.25 L each) using a peristaltic bomb. In addition, three negative filtration controls of 1.25 L each of distilled water were filtered. Each filter was stored in 600 μL of ATL buffer (Qiagen) and subsequently extracted with the Blood and Tissue kit (Qiagen), adjusting the volumes of AL buffer and ethanol to 600 μL and finally eluted with 100 μL of elution buffer. The elution was processed through the Zymo inhibitor removal kit following the manufacturer's instructions.

### 2.3 DNA metabarcoding libraries preparations and sequencing

Two different library sets were constructed for different sets of samples using two different primer pairs and conditions in order to specifically detect the macroinvertebrates present in the samples. Libraries were all prepared in triplicate. For the bulk samples library preparation, a fragment of the mitochondrial COI gene of around 460 bp (including primers) was amplified using the following primers: forward – BF3 (5′ CCHGAYATRGCHTTYCCHCG 3′) (Elbrecht et al., 2019) and reverse – BR2 (5′ TCDGGRTGNCCRAARAAYCA 3′) (Elbrecht & Leese, 2017). These primers also included the universal Illumina ligation sequences attached to their 5′ ends: forward universal tail (5′ ACACTCTTTCCCTACACGACGCTCTTCCGATCT 3′) and reverse universal tail (5′ GTGACTGGAGTTCAGACGTGTGCTCTTCCGATCT 3′). A variable number of nucleotides (three to six) were inserted between the primer and the universal Illumina ligation sequences to increase sequence diversity, which in turn leads to better results on Illumina machines and allows for a reduced spike‐in of ~5% PhiX (Elbrecht & Steinke, 2019; Wu et al., 2015). In the first amplification step, PCRs were carried out in a final volume of 25 μL, containing 2.5 μL of template DNA, 0.2 μM of the primers, 12.5 μL of 2× Multiplex PCR Master Mix (QIAGEN Multiplex PCR Plus kit), and ultrapure water up to 25 μL. The reaction mixture was incubated as follows: an initial denaturation step at 95°C for 5 min, followed by 30 cycles of 95°C for 30 s, 50°C for 90 s, 72°C for 30 s, and a final extension step at 68°C for 10 min. PCR products were purified using the Mag‐Bind RXNPure Plus magnetic beads (Omega Bio‐tek), following the instructions provided by the manufacturer.

For the eDNA library preparation, a fragment of the mitochondrial COI gene of around 191 bp (including primers) was amplified using the following primers: forward – fwhF2 (5′ GGDACWGGWTGAACWGTWTAYCCHCC 3′) (Vamos et al., 2017) and reverse – EPTDr2n (5′ CAAACAAATARDGGTATTCGDTY 3′) (Leese et al., 2020). These primers also included the Illumina ligation primer sequences attached to their 5′ ends and a variable number of nucleotides (four to six) in order to increase sequence diversity. In the first amplification step, PCRs were carried out in a final volume of 25 μL, containing 2.5 μL of template DNA, 0.3 μM of the primers, 12.5 μL of 2× Multiplex PCR Master Mix (QIAGEN Multiplex PCR Plus kit), and ultrapure water up to 25 μL. The reaction mixture was incubated as follows: an initial denaturation step at 95°C for 5 min, followed by 35 cycles of 95°C for 30 s, 50°C for 90 s, 72°C for 30 s, and a final extension step at 68°C for 10 min. PCR products were purified using the Mag‐Bind RXNPure Plus magnetic beads (Omega Bio‐tek), following the instructions provided by the manufacturer.

The oligonucleotide indices which are required for multiplexing different libraries in the same sequencing pool and the Illumina i‐5 and i‐7 sequences were attached in a second PCR round for both libraries. PCRs were carried out in a final volume of 25 μL, containing 1 μL of PCR product, 0.2 μM of the dual‐indexed primers, 12.5 μL of 2× Multiplex PCR Master Mix (QIAGEN Multiplex PCR Plus kit), 1x CoralDYE, and ultrapure water up to 25 μL. The reaction mixture was incubated as follows: an initial denaturation at 95°C for 5 min, followed by 20 cycles of 95°C for 30 s, 61°C for 30 s, 72°C for 42 s, and a final extension step at 68°C for 10 min. A negative control that contained no DNA was included in every PCR round to check for contamination during libraries preparation.

The libraries were verified on 2% agarose gels and purified using the Mag‐Bind RXNPure Plus magnetic beads (Omega Bio‐tek), following the manufacturer's instructions. The purification was carried out twice for the libraries amplified with the fwhF2/EPTDr2n primer pair, and just once for the BF3/BR2 primer pair. Finished libraries were pooled in equimolar amounts based on DNA quantification values determined using the Qubit 4 dsDNA HS Assay (Thermo Fisher Scientific) quantification. The pool was sequenced on a NovaSeq PE250 flow cell (Illumina) aiming for a total output of 100.000 reads per replicate. Raw sequencing data are available at NCBI SRA (Accession numbers: eDNA PRJNA1073854, bulk PRJNA1073752).

### 2.4 Bioinformatic analyses

Demultiplexed reads were processed through APSCALE v1.6.3 pipeline with the default values (e.g., max sequence Expected Error: 1, min and max amplicon length: ±10 bp length expected, min size to pool sequences: 4, OUT clustering: 97%, denoising alpha: 2, denoising min size: 8) (Buchner et al., 2022). Generated OTUs were taxonomically assigned against BOLD system v4 database (Ratnasingham & Hebert, 2007) using BOLDigger v1.5.6 pipeline (Buchner & Leese, 2020). Resulting OTUs were processed with Taxon Table Tools (Macher et al., 2020). For each sample, extraction replicate reads were merged, but consistency of PCR replicates was required, so only OTUs present in all three PCR replicates were retained for bulk samples, and only taxa present in at least two of the three PCR replicates were retained for eDNA samples. OTUs reads present in negative controls were subtracted from every sample. OTUs tables were condensed in a presence/absence taxa list.

The taxa list was processed in R v4.3.1. (R Core Team, 2023). We first looked for invasive or endangered taxa; then we filtered and removed OTUs that were not macrozoobenthos species and finally filtered and removed taxa not included in the IBMWP index.

### 2.5 Statistical analyses

All statistical analyses were performed in R v4.3.1. (R Core Team, 2023). A PERMANOVA test was run using the adonis2 function from the vegan v2.6 package to explore the effects of methodology and sampling location on the variance of species composition. A total of 99,999 permutations were performed, and the marginal effects of each variable were tested. Only the samples with both molecular methods applied were tested in order to have a balanced design. Spearman's correlation analyses were performed to analyze the correlation between molecular and morphological EQR values. A linear model of the correlation of the EQR values of each molecular method with respect to the morphological EQR was generated, and the differences were analyzed with respect to the intercept and the slope of the bisector. Ecological status differences between molecular and morphological approaches were tested with a Binomial and also with a Friedman test.

## 3 RESULTS

### 3.1 Species diversity

**FIGURE 2:**
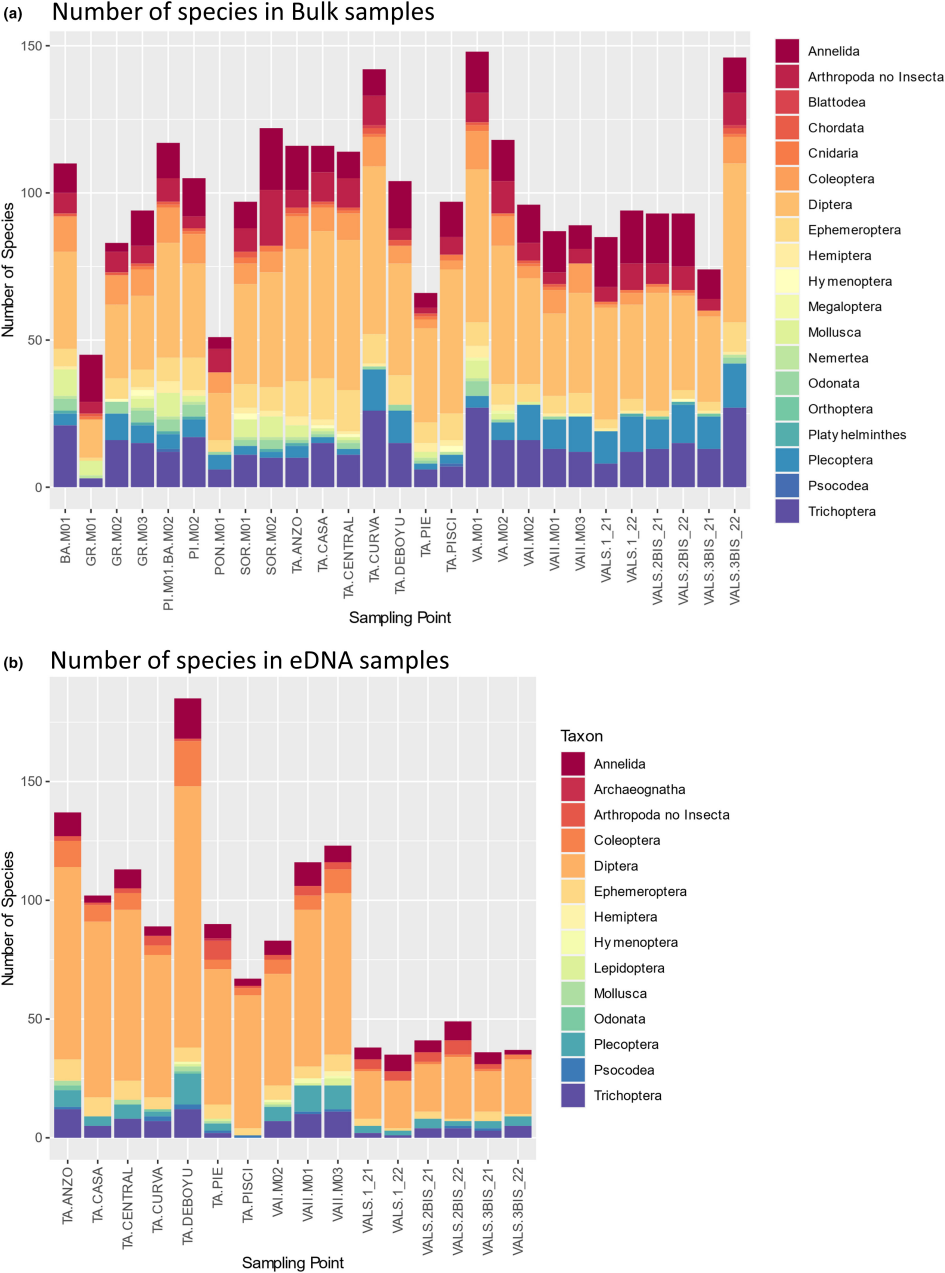
Species composition and number of species per sampling location. (a) Bulk samples. (b) eDNA samples. Chironomidae were the dominant IBMWP taxa in both bulk and eDNA samples. The second and third most dominant taxa varied between methods and were Acariformes and Oligochaeta in the bulk samples and Leuctridae and Simuliidae in the eDNA samples. In addition, several families (Anthomyiidae, Crambidae, and Thaumaleidae) were found in eDNA samples but not in bulk samples, suggesting sampling or amplification bias.

DNA metabarcoding of homogenized bulk samples resulted in a total of 460 detected species (643 OTUs) across all 27 samples with a mean value of 100 species per sample. A total of 360 species (405 OTUs) were detected by metabarcoding of water eDNA in 16 samples with a mean value of 84 species per sample (Figure 2). However, of all these species, only 160 OTUs were shared between both techniques (27.3% of the total), with 284 unique species detected in bulk samples (44.1%) and 184 in eDNA (28.6%) (Figure S1). The Jaccard dissimilarity in species composition between the two methodologies was 0.75 on average (Figure S2). The Class Insecta showed the highest species richness (bulk: 330 species, eDNA: 307) across all sites followed by Clitellata (bulk: 54, eDNA: 31) (Figure 1). PERMANOVA results showed that species composition was significantly related to the two variables analyzed: sampling location (*R* ^2^: .574, *F* = 2.3803, *p*‐value < .001) and type of sample used (*R* ^2^: .185, *F* = 11.4764, *p*‐value < .001).

### 3.2 Taxonomic resolution

A total of 2558 OTUs could not be identified to species level in the bulk samples, leaving 238 OTUs identified to genus level, 262 OTUs identified to family level, 378 OTUs to order level, 1600 to class level, and 60 to phylum level (Figure S3). In the case of the eDNA samples, 415 OTUs were identified at lower taxonomic level than species. Specifically, 182 OTUs were identified at genus level, 133 at family level, 43 at order level, 56 at class level, and 1 at phylum level (Figure S4).

### 3.3 Exotic/invasive and endangered/protected species detected

A total of 11 exotic species were detected by molecular tools. These species were grouped into the phylum Arthropoda (six species), Mollusca (two species), Platyhelminthes, Chordata, and Cnidaria (one species each) (Table 1). Despite being introduced species, only three of them (*Vespa velutina*, *Potamopyrgus antipodarum*, and *Pacifastacus leniusculus*) appear in the Spanish Catalogue of Invasive Alien Species (MAGRAMA, 2013b). As for the technique used, bulk proved to be the technique with the highest detection of exotic species, with a total of nine species, while eDNA was only able to detect four species. However, there are two species that were only detected with eDNA and not by bulk (*Acanthocyclops americanus* and *Drosophila suzukii*). The species that have been detected in the greatest number of samples were *Craspedacusta sowerbii* (62.96% of the samples), *P. antipodarum* (44.44% of the samples), and *P. acuta* (14.81% of the samples).

**TABLE 1:** Number of samples in which non‐indigenous species (NIS) were detected.

Non‐indigenous species (NIS)	Phylum (class)	Native distribution	Invasive?	Bulk (*n* = 27)	eDNA (*n* = 16)
***Pacifastacus leniusculus* (Dana, 1852)**	Arthropoda (Malacostracea)	North Western America	Yes MAGRAMA (2013b); Oliva‐Paterna et al. (2021)	3	2
*Chydorus brevilabris* *(*Frey, 1980)	Arthropoda (Branchiopoda)	North America	No	1	‐
***Acanthocyclops americanus* (Marsh, 1893)**	Arthropoda (Copepoda)	North America	Yes Alekseev (2021)	‐	3
***Vespa velutina*** **(Lepeletier, 1836)**	Arthropoda (Insecta)	South East Asia	Yes MAGRAMA (2013b)	1	‐
***Drosophila suzukii* (Matsumura, 1931)**	Arthropoda (Insecta)	South East Asia	Yes Fiel et al. (2014)	—	4
*Ceratophysella communis* (Folsom, 1897)	Arthropoda (Collembola)	South East Asia	No	1	—
*Girardia sinensis* (Chen & Wang, 2015)	Platyhelminthes	North America	No	1	—
***Onchorhynchus mykiss* (Walbaum, 1972)**	Chordata (Actinopterygii)	North Western America	Yes Oliva‐Paterna et al. (2021)	1	—
***Craspedacusta sowerbii* (Lankester, 1880)**	Cnidaria (Hydrozoa)	South East Asia	Yes Oliva‐Paterna et al. (2021)	17	—
***Potamopyrgus antipodatum* (Gray, 1843)**	Mollusca (Gastropoda)	New Zealand	Yes Oliva‐Paterna et al. (2021)	12	—
***Physella acuta* (Draparnaud, 1805)**	Mollusca (Gastropoda)	North America	Yes Oliva‐Paterna et al. (2021)	4	1

*Note*: *n*: Total number of samples processed by each method (in bold species declared as invasive in Spain).

The species *Rana iberica* (Boulenger, 1879), included in the Spanish List of Wildlife Species under Special Protection Regime (MAGRAMA, 2011), and classified as Vulnerable (VU) under the criteria and assessments of The IUCN Red List of Threatened Species in 2020, was detected in sample GR‐M02 by bulk sample metabarcoding (725 reads).

### 3.4 Freshwater quality status for river biomonitoring

**FIGURE 3:**
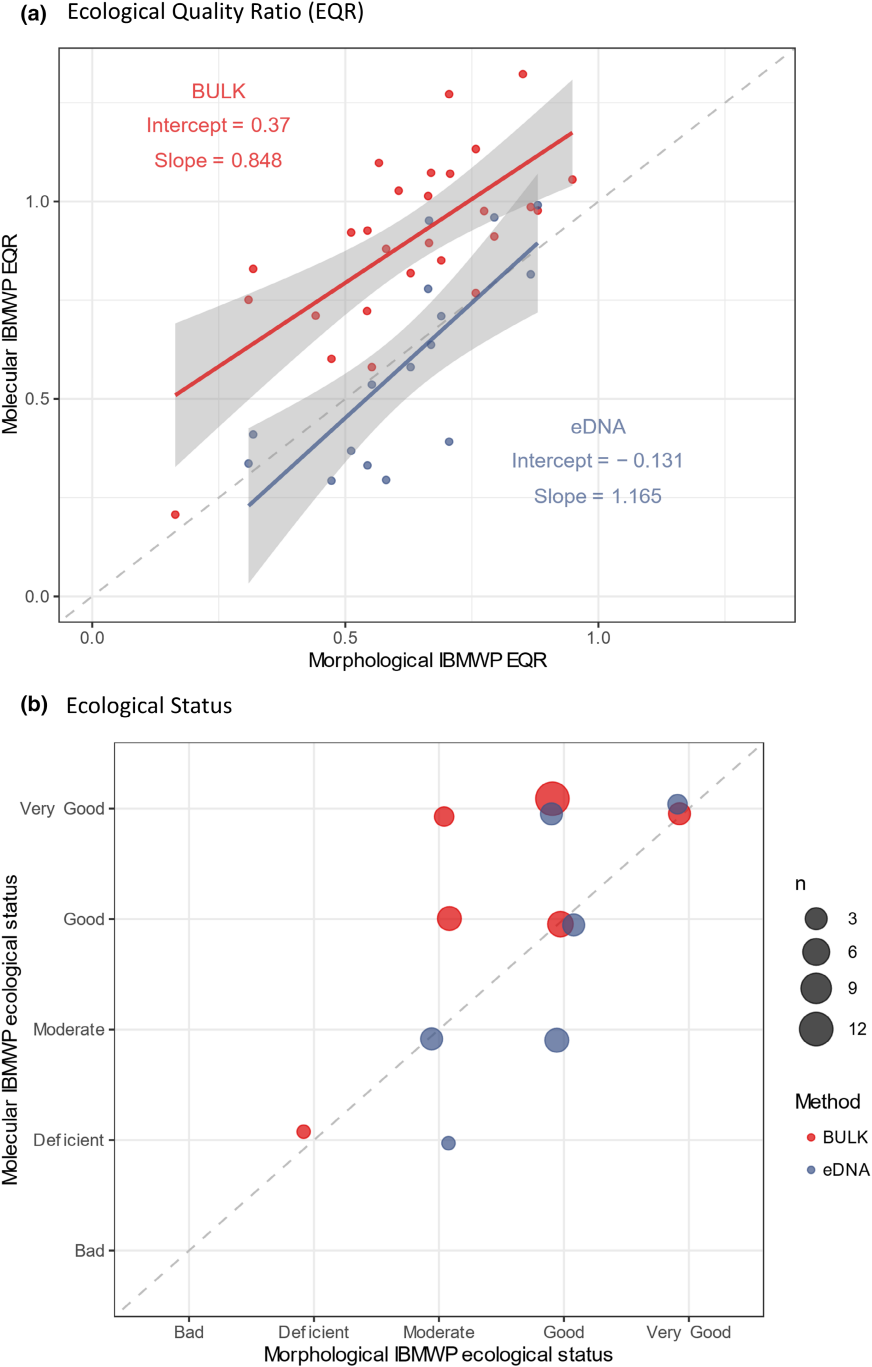
Comparison between (a) EQR and (b) ecological status obtained for the same sampling locations using different methods: Molecular (eDNA and bulk samples) and morphological. The linear model of EQR values for the eDNA samples does not differ significantly in either slope or intercept from the morphological method. However, the bulk samples have a significantly higher intercept which, as can be seen in Figurea, means that the EQR values are higher than those calculated for the same points using the morphological method. It can also be seen in (b) where the states inferred from the eDNA samples are around the visectrix (same value as morphological), but the bulk samples are mainly above it (better status than morphological).

The EQR values obtained by the two molecular techniques and those obtained by morphological identification were compared for the IBMWP index (Figure 3a). The results of Spearman's correlation analyses were significant for both the eDNA (rho = 0.773, *p*‐value < .001) and bulk samples (rho = 0.631, *p*‐value < .001). The linear model inferred for the eDNA samples shows no significant differences with either the slope (*p*‐value = .450) or the intercept of the bisector (*p*‐value = .543). In the case of the bulk samples, there is no significant difference between the slope of the linear model obtained and the bisector (*p*‐value = .377); however, there is a significant difference in the intercept (*p*‐value < .01) which is inferred as 0.37 points above the bisector origin. A total of 477 different IBMWP taxa were detected in the bulk samples (17.6 per sample) with an IASPT value of 6.04 (mean IBMWP value), while the morphological approach detected 359 IBMWP taxa (13.3 per sample) with an IASPT value of 5.64 per taxon. The Mann–Whitney *U* test performed to compare the IBMWP values between the two techniques gave results that met the significance level (W = 92410.5, *p*‐value < .05).

The ecological states inferred from the molecular data were compared with those obtained from the morphological data (Figure 3b). In the eDNA data, although there are variations in the categorization of the rivers between the two methods (eight equal states, three improved states, five worsened states), there is no significant tendency to infer a better or worse state than with the morphological data (*p*‐value binom test: .726, *p*‐value Friedman test: .479). However, in the bulk sample data, a higher percentage of differences (10 equal states, 17 improved states) are observed, and these tend significantly to a better value of ecological state than inferred from the morphological data (*p*‐value binom test < .001, *p*‐value Friedman test < .001).

## 4 DISCUSSION

### 4.1 Metabarcoding unraveling river's diversity: Native, exotic, and protected species

Achieving species‐level resolution by morphological identification of biodiversity in river kick samples is time‐consuming and requires expert taxonomists. Therefore, in this study, we have used molecular techniques such as DNA metabarcoding as a tool to identify species‐level diversity in a more efficient way. With this approach, we have been able to detect and identify a total of 628 different species from eDNA and bulk samples. However, only 160 species were detected with both methods, and the PERMANOVA analysis showed significant differences in species composition depending on the methodology used. The possibility that the genetic information in each type of sample differs could account for the variation in species composition found with each molecular technique. Bulk samples have been taken according to the official methodology of the ministry (MAGRAMA, 2013a), methodology adapted from (Barbour et al., 1998; Jáimez‐Cuéllar et al., 2002) which consists of multihabitat, stratified and semiquantitative sampling of a 100‐meter stretch of river with a Surber net. Similar sampling methodologies have been tested and have only managed to capture 50% of the species present in the stretch (Furse et al., 1981). On the other hand, the DNA captured in eDNA samples is not limited to the species present within the 100 meters sampled for the bulk sample but can provide DNA from several kilometers upstream of the sampling location (Deiner & Altermatt, 2014). In addition, DNA from terrestrial species is also detected in eDNA samples, which can be washed into the river by runoff or even from sewage treatment plants, which can ‘pollute’ the river with DNA from commercial species (Deiner et al., 2016; Inoue et al., 2023). Therefore, it is reasonable that the species composition of the genetic material contained in the two samples may vary greatly, although there could be other possible reasons for these differences, such as the fragment amplified by the eDNA primer being too small and not powerful enough to correctly assign at the species level (Yeo et al., 2020), or that using different primers for each sample type leads to different amplification biases (Leese et al., 2021).

DNA‐based molecular techniques are a reliable tool for diversity studies, and they are also widely used for species detection and identification of exotic and invasive species. Techniques such as qPCR or LAMP are used for the detection of single invasive species in aquatic environments (Carvalho et al., 2021; Cary et al., 2014; Keller et al., 2017; Peñarrubia et al., 2016; Sepulveda et al., 2020). But in addition to studying diversity as we have shown, the metabarcoding technique enables us to simultaneously identify several exotic and invasive alien species without additional work or cost. In this work, we have reported a total of 11 species whose native distribution area does not correspond to the Iberian Peninsula, eight of them considered as invasive species (Table 1). All of them correspond to species whose presence in our waters had already been proven by previous morphological and molecular studies, except for two species, the brachiopod *Chydorus brevilabris* and the springtail *Ceratophysella communis*. The species *Chydorus brevilabris* is a branchiopod crustacean with North American origin that has been reported in other European countries such as Belgium, France, Luxembourg, and the Netherlands (Soesbergen, 2020) and recently in the Asian country of Japan (Makino et al., 2023); however, its presence in the waters of the Iberian Peninsula was unknown. On the other hand, *C. communis* is a terrestrial springtail of Asian origin that has never been reported outside its native distribution. There seems to be a lack of documented impacts of these exotic species in the new environments where they have been introduced, and thus both species should be the focus of future genetic and morphological studies to confirm their presence and impacts in the region.

As noted by Fiel et al. (2014) and Sánchez and Arias (2021), invasive species such as the insects *Vespa velutina* and *Drosophila suzukii* are highly prevalent in the area, and it is therefore not unusual to find traces of genetic material in the samples. Similarly, the salmonid *Onchorhynchus mykiss* was introduced in Asturias for the purpose of salmonid aquaculture (Márquez et al., 2014). On the other hand, the invasive aquatic species of gastropod mollusks, *Potamopyrgus antipodarum* and *Physella acuta*, as well as the invasive decapod crustacean *Pacifastacus leniusculus*, are widely distributed and very abundant in our waters (Vedia et al., 2013). They were the most detected exotic species by molecular techniques during this study, appearing; in the case of *P. antipodarum*, in more than 40% of the samples analyzed. The presence of the invasive cnidarian species *Craspedacusta sowerbii*, an active predator of zooplankton that can change animal communities in the water bodies it colonizes (Smith & Alexander, 2008), in more than 60% of the samples is also thought to be the most relevant data found (Medina‐Gavilán & González‐Duarte, 2018). While its presence was known in a large area of the Iberian Peninsula and in the surrounding autonomous communities of Galicia and Castilla y León, its presence in the Principality of Asturias was unknown.

Finally, the exotic flatworm *Girardia sinensis* and the copepod *Acanthocyclops americanus* were only known from the central and western fringe of the Iberian Peninsula where no impacts on the ecosystem have been reported (Alekseev, 2021; Benítez‐Álvarez et al., 2023), corresponding, these records, to an increase in the knowledge of their distribution in this area. Since *A. americanus* can have less than 150 μm width, its presence in the sample can be lost when using a 500 μm Surber, and the processing method of the bulk samples likely favors the species' presence in eDNA rather than bulk.

As these tools are useful for the detection of exotic and invasive species, they are also suitable for the monitoring and detection of endangered and protected species. In this work, we report the detection of the Iberian frog (*Rana iberica*), a vulnerable species (IUCN, 2024) included in the Spanish List of Wildlife Species under Special Protection Regime (MAGRAMA, 2011). This species, endemic to the Iberian Peninsula, is mainly distributed in the northwestern region with stable populations in the Basque Country and the Central System (Pleguezuelos et al., 2002). It was detected within its known distribution area, in the surroundings of the Grandas de Salime reservoir (Asturias). This indicates that metabarcoding (if using adequate primer pairs) can be used as a tool for follow‐up campaigns for endemic and protected species in rivers.

Even with all the species detected, it was not possible to identify all the OTUs to species level. In the bulk samples, only 17.43% of the OTUs generated could be identified to species level; in the case of the eDNA samples, this percentage rose to 49.4%. This lack of taxonomic resolution can have several causes, such as the lack of sequences in the reference databases that prevent the taxonomic assignment of those species that are not currently found in the databases (Csabai et al., 2023; Fueyo et al., 2024; Weigand et al., 2019). But it may also be due to the presence of NUMTs (nuclear copies of mitochondrial genes) or heteroplasmy, as they can provide OTU diversity that does not translate into real species diversity (Ožana et al., 2022; Schultz & Hebert, 2022). The lack of sequences in the reference databases of these variations or of reliable systems integrated into the bioinformatic pipelines of metabarcoding prevents the correct filtering of these variations. This results in the generation of spurious OTUs, many of which are identified at lower taxonomic levels (Anderson & Leite, 2012; Porter & Hajibabaei, 2021). These limitations in taxonomic resolution have little impact on the management of invasive, protected species or species of regional interest, as most of these species have sequences in the databases due to their importance. The number of OTUs that are not identified to species level and are not the result of artifacts, heteroplasmy, or NUMTs can be gradually reduced by increasing the reference databases. To this end, there are many sequencing projects such as the International Barcode of Life (IBOL).

### 4.2 Metabarcoding for biomonitoring the ecological status of river ecosystems

In addition to diversity studies and the detection of exotic and endangered species, molecular techniques for biomonitoring the ecological status of river ecosystems are being proposed (e.g., Fernández et al., 2019; Keck et al., 2022; Mortágua et al., 2019). Our results find significant correlations between the EQR values of each molecular technique with respect to those obtained by morphological identification for the IBMWP index showing the potential of these techniques for application in biomonitoring. However, the intercept of the linear model of the EQR values of the Bulk samples has a significantly higher intercept than expected if the values were similar to those obtained by morphological identification. This indicates that the metabarcoding of bulk samples would be detecting more taxa or taxa with higher index values. In principle, metabarcoding of bulk samples is expected to have a higher detection rate (e.g., Kuntke et al., 2020), as it is able to detect species from elements that do not easily allow their morphological identification, such as eggs, pupae, feces, and parts of an individual (legs, abdomen, guts, etc.), and it is even possible to detect the presence of a species by the presence of its DNA in the stomach contents of its predators (de Sousa et al., 2019). However, there is a lack of studies that go into detail to check all the false‐negative and false‐positive detections to differentiate ecologically relevant results from artifacts of the technique, as these will translate into changes in EQR values and hence inferred ecological status.

Only 50% of the statuses inferred from the eDNA results coincide with those inferred from the morphological data when the metabarcoding EQR values are converted into ecological status, but there are no significant trends toward poorer or better status. However, the bulk samples not only had 63% of samples with a different inferred status, but these tended to be significantly higher than those inferred from the morphologically detected families. These results clearly show that metabarcoding results cannot be directly applied on top of the current indices to infer ecological status since, among other things, the current indices are designed and calibrated with samples processed using morphological identification approach.

Both the difference in detectability and the differences in EQR and inferred ecological status are not a problem in the final implementation of these techniques for biomonitoring, as they still have to go through a long process of optimization, fine tuning and intercalibration before they are ready for final testing for biomonitoring as when the currently used morphological techniques were standardized (Birk et al., 2018; Friberg et al., 2006; Furse, Hering, Brabec, et al., 2006). During this process, known problems in the application of these techniques should be corrected, such as the lack of sequences in the reference databases (Fueyo et al., 2024), and intercomparison experiments should be carried out to determine the replicability of the technique (Blackman et al., 2019). In addition, a more detailed study of the differences in detection between the metabarcoding and morphological identification and their possible causes is needed, as this can directly influence the results of the ecological status calculation.

### 4.3 Consideration for management

The scalability provided by molecular techniques promises to facilitate biomonitoring programs for diversity in the Iberian Peninsula. This, together with the progress in the study of other species groups (Apothéloz‐Perret‐Gentil et al., 2021) or the advancement of the eDNA technique with other types of samples (Garrett et al., 2023; Macher et al., 2023), shows the potential of this technique to achieve integrated monitoring of the global diversity of ecosystems in the future. As we have seen in this study, the multiapproach properties of metabarcoding not only position it as a pathway for studying diversity in rivers but also as a technique for monitoring endangered and invasive species and inferring the ecological status of rivers. All of this is within a single analysis. Nevertheless, the wide variation in results between the different methods casts doubt on their interpretation for use in management. Previous studies have shown that results from bulk samples are more similar to eDNA results than to those from the morphological method, which is the gold standard (Gleason et al., 2020). In addition, certain characteristics of bulk samples make them more reliable than eDNA for species identification. These are:

- The ability to use longer genetic markers, as the DNA in bulk samples is expected to be less degraded than in an eDNA sample. This allows more genetic information to be provided when identifying sequences, giving more confidence in species identification, although previous studies show that fragments larger than 200 bp are sufficient (Yeo et al., 2020).
- The possibility of using more degenerate primers as there is less risk of unspecific amplification in bulk samples. This makes it easier to avoid false‐negative detections due to primer bias. Have a higher concentration of DNA per individual in the sample as it is extracted directly from tissue. Potentially ensure the presence of DNA from all species in all extraction and PCR replicates.
- Much better delimitation of the spatial scale of the study, as eDNA can be washed to the sampling site from miles upstream of the river or from the land with runoff water (Deiner et al., 2016). This is a major problem when this DNA comes from sewage water as it may contain DNA from species that are not found in the river but rather species for human consumption or from aquariums (Inoue et al., 2023).

Yet eDNA also has its advantages compared to bulk samples in terms of species identification as it can detect species smaller than 500 um which is the pore size of Surber nets, and the primer bias is different from the primers used in bulk samples. We consider it necessary to take all these characteristics into account when using the results for management decisions.

Further efforts are needed to optimize and refine these two molecular techniques for use in diversity and/or biomonitoring studies. This includes species sequencing, development of informatics tools integrated into metabarcoding pipelines to detect and remove sequencing artifacts and NUMTs, development of better primers, implementation intercalibration projects, etc. Still, these DNA‐based techniques continue to be useful alongside morphological identification. They allow higher taxonomic resolution, detect cryptic species, identify species that do not have the morphological characteristics that define them in good condition or for which there are no identification keys, identify species at different stages of development, and have a second molecular confirmation of identifications, reducing human error.

The present study serves as a further step in implementing these molecular techniques for riverine biomonitoring in the Iberian Peninsula, one of many that need to be taken before a robust and reliable technique is available for routine use. The findings of this study serve as a baseline for future investigations into species diversity within this region.

## AUTHOR CONTRIBUTIONS

**Álvaro Fueyo:** Conceptualization (equal); data curation (equal); formal analysis (supporting); funding acquisition (equal); investigation (lead); methodology (equal); project administration (equal); resources (equal); visualization (lead); writing – original draft (lead). **Omar Sánchez:** Conceptualization (equal); data curation (supporting); investigation (equal); resources (equal); writing – original draft (equal). **Carlos Carleos:** Data curation (equal); formal analysis (lead). **Amando Escudero:** Resources (equal). **Javier Cordón:** Resources (equal). **Javier Granero‐Castro:** Funding acquisition (equal); project administration (equal); supervision (supporting). **Yaisel Juan Borrell:** Conceptualization (equal); supervision (lead); writing – review and editing (lead).

## CONFLICT OF INTEREST STATEMENT

The authors declare no conflicts of interest.

## Supporting information


Appendix S1.

Figure S1.

## Data Availability

The data that support the findings of this study are openly available in NCBI SRA database at https://www.ncbi.nlm.nih.gov/sra, reference number for eDNA data PRJNA1073854 and bulk data PRJNA1073752.
