# Supplementary material for: Unlocking rivers' hidden diversity and ecological status using DNA metabarcoding in Northwest Spain
Source: Ecol Evol. 2024 Aug 1;14(8):e70110. doi: 10.1002/ece3.70110 (PMC11294579; doi:10.1002/ece3.70110)
Supplement: Supplementary file 2 — Figure S1. [file ECE3-14-e70110-s002.docx]

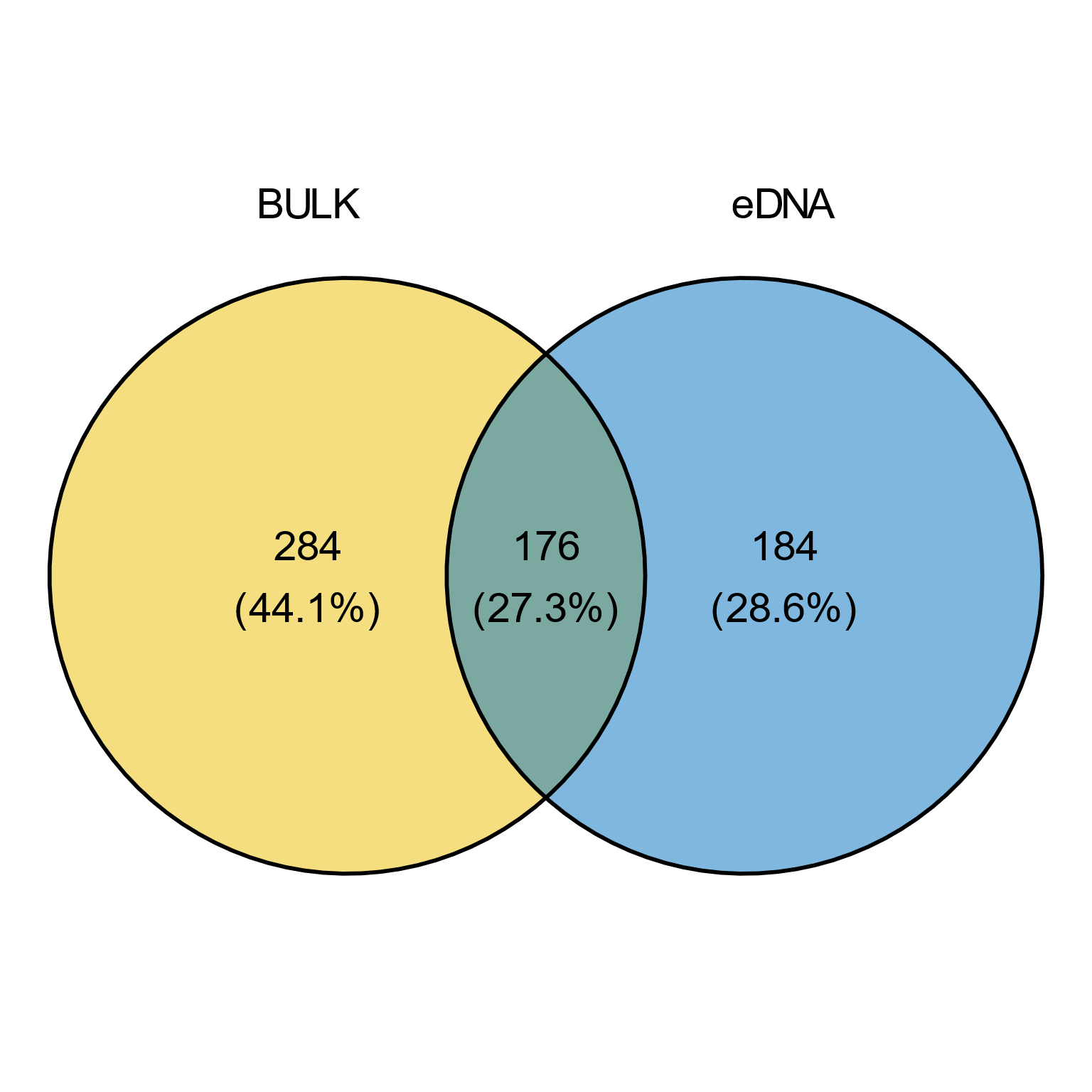
**Supplementary Information**
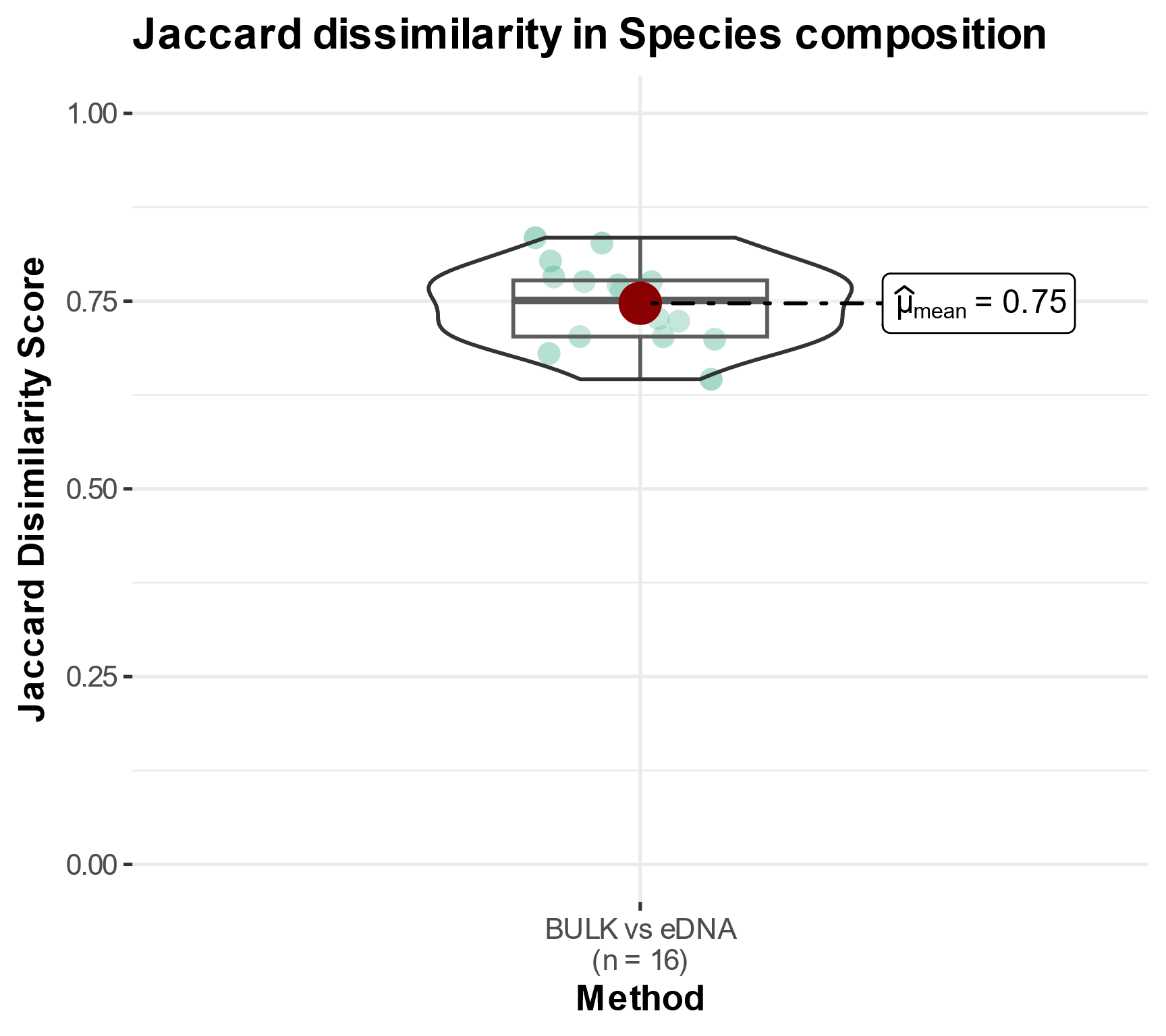


Figura S1. Venn diagram showing the species diversity shared between Bulk and eDNA samples.

Figura S1. Venn diagram showing the species diversity shared between Bulk and eDNA samples.

Figure S2. Jaccard dissimilarity scores between eDNA and Bulk samples collected at the same sampling point.

Figure S3. Taxonomic resolution of OTUs generated in BULK samples.


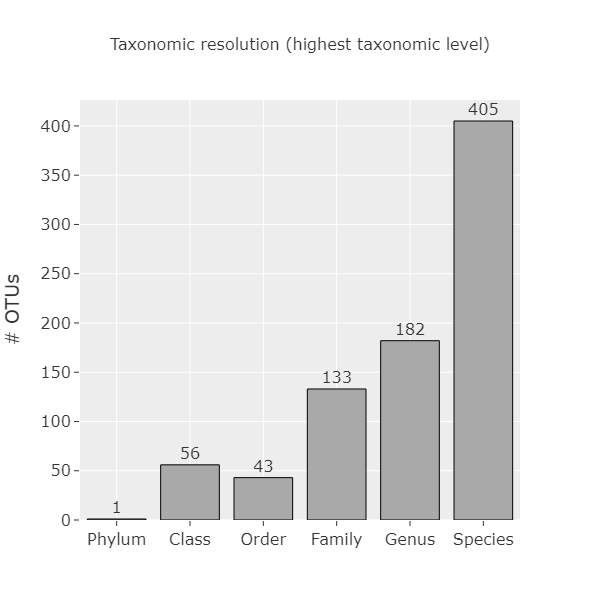

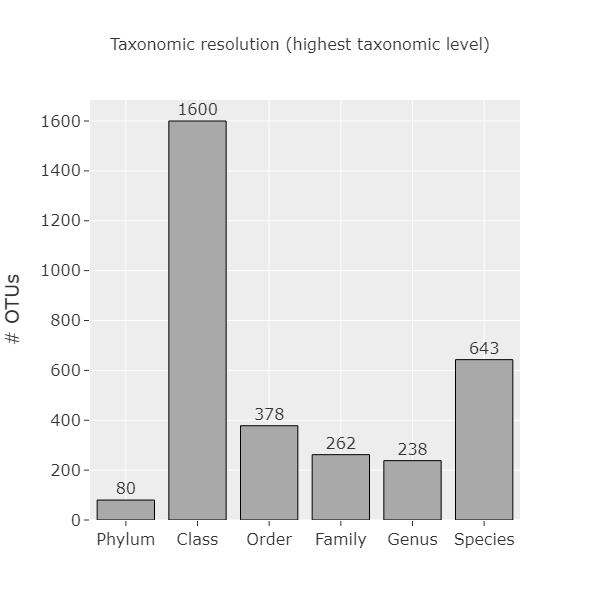


Figure S4. Taxonomic resolution of OTUs generated in eDNA samples.
